# The Theatre of Surgery: The Ethical and Legal Implications of Live Surgeries

**DOI:** 10.7759/cureus.66351

**Published:** 2024-08-07

**Authors:** Satvik N Pai, Naveen Jeyaraman, Madhan Jeyaraman, Sankalp Yadav

**Affiliations:** 1 Orthopaedics, People's Education Society (PES) University Institute of Medical Sciences and Research, Bengaluru, IND; 2 Orthopaedics, ACS Medical College and Hospital, Dr. MGR Educational and Research Institute, Chennai, IND; 3 Medicine, Shri Madan Lal Khurana Chest Clinic, New Delhi, IND

**Keywords:** law, ethics, medical conferences, live surgical broadcasting, live surgery

## Abstract

Surgical training has long emphasized learning through direct observation, allowing young surgeons to gain practical insights from experienced surgeons. The advent of live surgical demonstrations has extended this learning method, providing real-time broadcasts of surgeries to wider audiences. Live surgery is a surgery that is broadcasted in real time to an audience. While live surgeries offer substantial educational benefits, enabling the rapid dissemination of advanced surgical techniques and reducing the learning curve for surgeons, they also raise critical ethical and legal questions. Concerns include potential compromises in surgical outcomes due to increased pressure on surgeons, the ethical implications of patient consent, privacy issues, and the ambiguity of accountability when complications arise. In India, these concerns have intensified following a patient's death during a live surgery, prompting legal scrutiny and a Supreme Court petition seeking to regulate the practice. This article delves into the multifaceted debate surrounding live surgeries, examining both their educational value and the ethical, legal, and practical challenges they pose. We aim to provide a comprehensive overview that will inform readers about the complexities of live surgeries, spread awareness, and stimulate further discussions on this evolving practice.

## Editorial

One learns more from watching the masters than one does from reading books. This statement has always stood true when it comes to surgical training. While one can acquire theoretical knowledge by reading books and journals, there is a lot to be learned beyond these by observing a surgery or procedure being performed. This was how surgical training had been for many decades and continues to be to a large extent. With technological advancements, a mode of learning came about that expanded this method and enabled it to reach many more individuals. This was the start of live surgical demonstrations.

What is a live surgery? It is simply a surgery that is broadcasted in real time to an audience. Well, it is not very different from a regular surgery. The surgeon performs surgery on a real-life patient suffering from a real illness. The surgery is intended to be therapeutic and is clinically indicated. The surgeon performing the surgery is not a novice in training, but a qualified, experienced surgeon. The surgery is not an experimental one, but something the surgeon has experience in performing. Everything sounding like a normal surgery? Well, it is. Apart from one thing, the entire surgery is video recorded, and the live feed is displayed at the same/distant venue where the audience members are learning from the demonstration. The audience is usually filled with doctors of the same specialty or trainees in the particular specialty. Sometimes, the audience may ask the surgeons certain questions or clarifications, which the surgeon answers or demonstrates in real time. If it is otherwise so similar to a normal surgery, then why this debate and controversy? That is what we are going to delve a little deeper into.

The ethics argument

While live surgeries have been occurring at various medical conferences and workshops for a few decades, there have always been a fraction of individuals, both within and outside the medical fraternity, who believe that live surgeries are unethical. We can boil down the entire ethical debate to two questions: does live surgery compromise the surgical outcome and does the benefit outweigh the risk?

First, let us get into the benefits of live surgeries, because that is straightforward and agreeable to most. Live surgeries are an excellent opportunity for learning. It provides a lot of practical tips and guides for the performing of surgeries. Sharing of such live surgical demonstrations helps the spread of knowledge and expertise, thereby enabling surgeons to be updated about advances in the field and in turn provide better outcomes for their patients. Watching the masters do it correctly probably avoids experimentation by each surgeon and shortens the learning curve. As surgeons are upskilling themselves, patients and society are benefiting as well. It is also a means for efficient sharing of knowledge. For example, if a novel surgical approach is being performed in the USA, maybe a handful of surgeons from India would ever visit that center in the USA to learn that surgical procedure. Having a live surgical demonstration of that surgery would mean hundreds of surgeons in India could witness and learn a surgical procedure that they otherwise might not have done. Not only does it mean it is economical, but it also means that the knowledge spreads to a much wider audience than it would otherwise.

Now, let us see the point of the detractors. Their main contention is that live surgeries compromise the surgical outcome and health of the patient. However, how does live surgery compromise surgical outcomes if it is being performed by an experienced surgeon on a patient who really requires it? Does having the camera on a surgeon while performing a surgery impact the outcome? The answer is probably yes, and there are three primary reasons for it. Firstly, the surgeon is under pressure while performing the surgery. While a surgeon is under certain pressure when doing any surgery, recorded or not, the pressure is increased while performing a live surgery. With every move of the surgeon being watched by several of his peers, it is not only the health of the patient on the line but also the reputation of the surgeon. Some argue that surgeons are not new to being watched during surgery. Apart from the operating theatre (OT) staff, anesthetists, and assistant surgeons present, in teaching institutes, there are undergraduates, postgraduates, fellows, or even colleagues watching the surgical procedure. However, being watched by juniors, or known colleagues who have worked with you, is different from a live surgical demonstration at a conference. With live surgeries, most members of the audience are watching the surgeon for the first time and are going to be forming their opinions on the surgeon based almost entirely on the few minutes/hours of the live surgery. The being watched factor may also play a role when it comes to the decision-making of the surgeon during the surgery. There have been instances where the surgeons have refused to acknowledge a complication or change the plan of surgery after facing difficulty during a live surgery, something they probably would have been earlier to acknowledge and change their plan with if it were not a broadcasted surgery.

Secondly, the requirements placed on the surgeon are increased during live surgery. Apart from having to perform the actual surgical steps, the surgeon has to narrate what they are doing, demonstrate certain findings or steps specifically ensuring that it is visible to the camera, and answer questions put forth to him. Now, one can argue that all of these requirements are also placed on any surgeon performing surgeries with students or junior surgeons watching the surgery, which is valid. However, that is usually in a familiar setting. Live surgeries are often performed by invited faculty at centers where they do not normally operate. This means a new OT, unfamiliar OT staff, unfamiliar surgical assistants, and maybe even different instruments than what they are used to. These increased demands placed on the surgeon may lead to errors, which can cost the patient dearly.

Thirdly, the accountability for live surgeries remains a little ambiguous. When it is invited faculty performing the surgeries, the patient being operated on would often never have even met the operating surgeon. The patient is admitted under a surgeon at the hosting institute but is operated on by a different surgeon. Since the invited faculty leaves after the surgery, the post-operative follow-up is again left to a surgeon who has not performed the surgery. In the event of a surgical complication occurring, who would be responsible, the operating surgeon or the surgeon under whom the patient is admitted?

The legality

In India, there is no law permitting or prohibiting live surgeries. Does this make live surgeries legal or illegal? It would be the former, and so, thousands of live surgeries have been performed over the past few decades. However, recently, the legality has come under the scanner. This debate entered into the public domain after a patient undergoing a live surgical demonstration passed away during the live surgery. The incident occurred during a live surgical demonstration of a laparoscopic liver resection surgery being performed at All India Institute of Medical Science, New Delhi, by a Japanese surgeon as part of the 23rd annual conference of the Indian National Association for Liver Studies. During the surgery, the surgeon encountered bleeding, which was not able to be controlled sufficiently. Despite suggestions by colleagues that they convert it to an open surgical procedure, the surgeon persisted with the laparoscopic technique. Only after seven hours of the surgery was the laparoscopic surgery abandoned. At that point, the live feed to the audience was stopped, and the patient was shifted to the intensive care unit, where the patient passed away 90 minutes later [1]. This incident brought to the forefront the ethical debate on live surgeries and also multiple questions on its legality.

In 2023, a petition was filed in the Supreme Court of India seeking to ban or regulate live surgeries at medical conferences. The petitioner claimed that advertising sponsorship and professional showmanship overshadow the true purpose of these broadcasts, healthcare facilities showcase their capabilities, surgeons flaunt their skills, and companies promote their products all at the expense of patient safety [2]. The Supreme Court has asked the National Medical Commission (NMC) and the central government to deliberate on the topic and give its responses [3]. The NMC has made a panel that is deliberating over the same. After receiving the suggestions by the central government and the NMC, the Supreme Court will pass a verdict on the petition. That verdict will clarify the legal standing on live surgeries. Until that though, live surgeries continue to be legal.

When we look at other countries, we find that live surgeries continue to be performed in most other countries as they do not violate any specific laws. However, many medical association bodies have deliberated over this practice, and some have stopped the practice of live surgeries entirely. The Japanese Society of Thoracic Surgeons was among the earliest to ban live surgeries after a patient died during one such live demonstration in 2006. Other associations that have taken a similar stance in banning live surgeries are the Japanese Urology Association, the American College of Surgeons, the American College of Obstetricians and Gynaecologists, and the American Association for Thoracic Surgery. In India, too, some associations have clearly laid down a policy of having no live surgeries in their conferences. The Association of Breast Surgeons of India (ABSI) and the All India Ophthalmic Society have declared such a policy [3].

Privacy and confidentiality

There are certain other aspects of live surgeries that can also have legal ramifications. The privacy of the patient is something that has to be safeguarded. As far as possible, the patient's identity must not be revealed. There have been instances where the surgeon giving a background about the case reveals the patient's name, which is not required. The persons recording the video must consciously try to avoid revealing the face of the patient. If this is not ensured, it might become grounds for the patient to sue the surgical team for violating their right to privacy. Another aspect that organizers of such events and surgeons performing the live surgeries must check is whether the surgeon is licensed to perform a surgical procedure in that geographical location. Live surgeries often have expert surgeons visiting from other states or countries to perform the surgery. They must ensure that they are registered as medical practitioners in the state and country where the surgery is being performed. If not, they require special permission/temporary registration from the medical council to be obtained beforehand. One more legal aspect is that relating to consent. It must be informed to the patient that the surgery will be broadcasted, and ideally, consent for the same must be obtained from the patient. If consent is not obtained, it would make it an ethically objectionable activity. One of the factors that safeguarded the surgeons in the case mentioned in the previous paragraph was the fact that the consent for the live broadcast of the surgery and the risk involved in the surgery were informed to the patient prior to the surgery [1].

Authors' perspective

We do not agree with a lot of points raised in the public interest litigation before the Supreme Court. We believe that live surgical demonstrations are a great learning opportunity, and most conferences are doing it for purely educational purposes. If it is occasionally done with ulterior motives of showcasing a product or surgeon's professional skill, those types of events should be discouraged, but it would not be appropriate to ban all live surgeries for that reason. The petition also claims that performing a live surgery while answering questions is like Virat Kohli having to bat and commentate at the same time [4]. This we think is an absolute misrepresentation. During any surgical procedure, it is commonplace for the assistant surgeon or student to ask the surgeon questions during the surgery, and the surgeon does respond. The persons asking the question are aware of the critical moments of the surgery when they should let the surgeon focus with undivided attention. The surgeons themselves are aware of when they can answer questions and at what steps they would not be able to answer any questions. Virat Kohli has to make a split-second decision when the bowl is delivered, but there is no such millisecond time restriction on a surgeon, and if it is a critical step requiring immediate action, the surgeon can choose to answer the questions later.

A study performed by Rocco et al. [5] analyzing the rate of complications in live surgeries found that the incidence of complications was low and at par with the expected rate of complications considering the complexities of the surgeries that were performed. This suggests that live surgeries do not adversely impact the performance of the surgeon. However, we would need a lot more of such studies to conclusively say if live surgeries hamper surgical outcomes. Surgical demonstrations are an invaluable form of knowledge sharing and skill acquisition for surgeons, which will help medical advancement and patient outcomes. That being said, justice must be done to the patient. Consent must be taken from the patient, and if the organizers of the conferences are monetarily benefiting from the conference, we would encourage the benefits to be extended to the patient in the form of decreasing the cost of the surgeries or performing the surgery free of cost. In order to further decrease some of the practical difficulties of live surgeries, we recommend that whenever possible, surgeries should be performed at the base of the operating surgeon and broadcasted to the venue, rather than have the surgeon operate at the host venue, which would be an unfamiliar setup, instruments, and OT personnel for the surgeon. As far as accountability is concerned, the operating surgeon and the admitting team must be aware that both of them would share liability if complications arose. Live surgeries must be performed only after all the parties are aware and accepting of this risk.

We have also considered alternatives to live broadcasts. Pre-recorded surgical demonstrations being displayed at the conference, with the operating surgeon being available to answer questions from the audience during the demonstration, may be a viable alternative. It would enable knowledge sharing and clarification of doubts. It would also eliminate the wastage of time due to technical issues that often happen in the recording or broadcast. We understand that it would not be quite like a live surgical demonstration, as it would not be real time and not allow the surgeon to be able to show certain points the audience might want clarifications on. The biggest advantage we think would be that it eliminates the pressure on the surgeon to deliver a near-flawless surgery in that single attempt while being watched in real time. We believe that this option should be preferred in most cases, and live surgical demonstrations should be reserved for particular cases where it would be significantly better to have a live discussion and real-time decision-making is a significant part of the surgery. Live surgical demonstrations must be reserved only for the most experienced surgeons who themselves feel comfortable performing live surgeries, and even to them, the choice of rather doing a recorded demonstration with live commentary should be offered.

Conclusions

With the rapidly increasing number of live surgeries, the clarification on its legality is going to be vital in shaping the future of live surgeries. It is going to be interesting to see the recommendations of the National Medical Commission in this regard and the guidelines that might be introduced in the future. We believe that live surgeries have a lot to offer in terms of knowledge sharing and acquisition of skills for surgeons, but it does come with its own ethical and practical problems. We suggest that pre-recorded surgical demonstrations with live commentary and discussions with the operating surgeon may be an alternative that preserves most of the benefits of live surgeries, which also make it safer. Technological advancements have allowed for live surgeries to be a reality and increase their reach. We believe that in the course of time, further technological advancements will also make it safer. For now, it is imperative that clear guidelines or clarifications be provided by policymakers after taking into consideration all the ethical and practical aspects of live surgeries.
